# Tick-borne pathogens in neotropical animals in Trinidad, West Indies

**DOI:** 10.1186/s13071-022-05184-z

**Published:** 2022-02-19

**Authors:** Candice Sant, Devon Seunarine, Nadine Holder, Krystal Maharaj, Melanie Vaughan, Shimon Harrus, Ricardo Gutierrez, Yaarit Nachum-Biala, Gad Baneth, Roxanne Charles, Patricia Pow-Brown, Rod Suepaul, Karla Georges

**Affiliations:** 1 School of Veterinary Medicine, Faculty of Medical Sciences, The University of the West Indies, St Augustine, Trinidad and Tobago; 2 Koret School of Veterinary Medicine, The Hebrew University of Jerusalem, Rehovot, Israel

**Keywords:** *Anaplasma*, *Ehrlichia*, Hunters, Neotropical animals, *Theileria*, Tick-borne pathogens, Trinidad

## Abstract

**Background:**

Ticks are important vectors of many pathogens that have contributed to the morbidity and mortality of humans and domestic animals worldwide. Wildlife species have also been implicated as reservoir hosts of a variety of tick-borne pathogens. The objective of this study was to determine which tick-transmitted pathogens were present in the animals harvested from the forest in Trinidad for human consumption.

**Methods:**

Thin blood smears from 43 neotropical animals were examined microscopically for tick-borne pathogens. Additionally, DNA extraction and PCR amplification of the *16S rRNA* gene were used for amplification of *Anaplasma* and *Ehrlichia* while the *gltA* gene was used for *Bartonella*, and *Rickettsia* spp. and the *18S rRNA* gene for *Babesia*, *Hepatozoon* and *Theileria* species.

**Results:**

Pathogen DNA was amplified from four samples (a deer, collared peccary and two agoutis). Sequencing of the amplified products from the deer and collared peccary revealed 99.8% homology to *Anaplasma bovis* and 98.8% homology to *Ehrlichia canis*, respectively. Sequences from two agoutis revealed 90.4% homology to *Theileria* spp. DNA of *Hepatozoon* spp., *Bartonella* spp. *Babesia* spp. and *Rickettsia* spp. was not detected in any of the screened samples. An incidental finding in this study was the presence of bacteria in the blood of animals.

**Conclusions:**

The results indicate that the DNA of tick-transmitted pathogens is present at a frequency of about 10% in the study population and suggests that neotropical mammals may serve as a source for the potential transmission of tick-borne pathogens to domestic animals and humans. In addition, physicians and hunters should be aware of the symptoms associated with zoonotic tick-borne pathogens so that these infections can be recognised, diagnosed and treated promptly. Bacteria present in carcasses can pose a food safety hazard and hunters should be trained in proper harvesting and handling of carcasses.

**Graphical Abstract:**

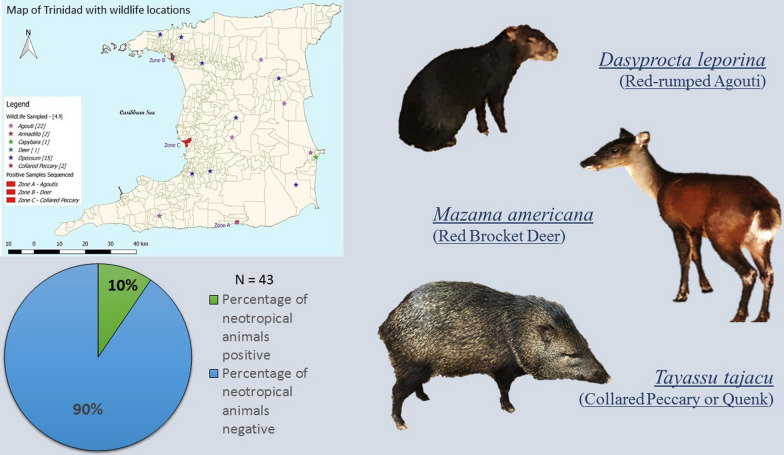

## Background

Ticks are considered to be the second most efficient arthropod vectors of pathogens after mosquitoes [1]. Trinidad is the southernmost island in the Caribbean, located at 10.69°N, 61.22°W, has a tropical climate, with similar flora and fauna to the South American mainland, and is ideal for the year-round proliferation of ticks and other arthropod vectors. *Argas*, *Ornithodoros*, *Amblyomma*, *Dermacentor*, *Haemaphysalis*, *Ixodes* and *Rhipicephalus* spp. have been recovered from humans, bats, canids, felids, equids, ruminants, rodents, marsupials, birds, amphibians and reptiles in Trinidad [2].

Human interaction with wildlife occurs frequently during the hunting season. Hunters overnight in forest camps in close proximity to their dogs. Neotropical animal carcasses are placed in bags that are carried on the hunter’s back thereby further increasing the risk of tick transmission from the carcasses. Hunters also use a “sentry” style of hunting whereby they sit for several hours on a perch in the forest, which places them at increased risk of being bitten by questing ticks, other arthropods and wild animals.

Wildlife have been implicated as reservoir hosts for tick-transmitted pathogens [3–5]. Most infected wildlife species are asymptomatic or may exhibit mild symptoms, which may be difficult to detect [6]. Factors such as deforestation, illegal logging activities, hunting and farming including the rearing of wild animals have resulted in increased contact of humans and domestic animals with wild animals. Indigenous neotropical mammals such as the agouti (*Dasyprocta leporina*), deer/red brocket deer (*Mazama americana*), Lappe/spotted paca (*Cuniculus paca*), manicou/black-eared opossum (*Didelphis marsupialis insularis*), tattoo/nine-banded armadillo (*Dasypus novemcinctus*) and wild hog/quenk/collared peccary (*Tayassu tajacu*) are popular game animals in Trinidad. These animals are also reared in wildlife farming facilities, which are becoming established with increasing frequency [7]. These popular game species are known to harbor ticks that are capable of transmitting diseases between animals [8].

*Ehrlichia* spp. and *Anaplasma* spp*.* are small, gram-negative obligate intracellular bacteria belonging to the family Anaplasmataceae that are transmitted through the saliva of an infested tick, feeding on a host. They infect endothelial cells and leucocytes, specifically granulocytes, monocytes and macrophages of the mammalian hosts [9]. *Ehrlichia chaffeensis* and *E. ewingii* can affect humans [10]. *Ehrlichia canis*, a common tick-borne pathogen present in the canine population of Trinidad and Tobago [11], is transmitted by the brown dog-tick, *Rhipicephalus sanguineus* (s.l.). Suspected cases of human ehrlichiosis due to species closely related to *E. canis* have been reported in Venezuela and Costa Rica [12, 13]. Common *Anaplasma* spp. of domestic animals are *A. marginale*, *A. centrale*, *A. ovis*, *A. bovis*, *A. phagocytophilum* and *A. platys*. There is a dearth of published data available on infections with *Anaplasma* spp. of neotropical animals.

*Bartonella* spp. are gram-negative intracellular bacteria of zoonotic importance and are mainly transmitted by blood-sucking arthropod vectors such as fleas, ticks, lice and flies [14–17]. *Bartonella henselae* and *B. clarridgeiae* have been reported in domestic cats in Trinidad and Tobago using molecular methods [18]. *Rickettsia* spp. are gram-negative bacilli and are transmitted by fleas, lice and ticks [19]. Reservoir hosts of *Rickettsia* spp. include the opossum and capybara [20, 21]. *Amblyomma cajennense* and *R. sanguineus* (s.l.) tick vectors as well as the opossum and capybara are present in Trinidad [2, 22]. The opossum and capybara can thus potentially serve as a source of *R. rickettsii* infection to humans [20, 21]. There are no published reports on the presence of *Rickettsia* spp. in Trinidad.

*Babesia* spp. and *Theileria* spp. are protozoan parasites transmitted by ixodid ticks. Piroplasms have been identified in the blood of armadillos, opossums, agoutis, pacas, red brocket and grey brocket deer in the rainforest in French Guiana [23]. Reports based on phylogenetic analyses of the *18S rRNA* gene of piroplasms obtained from an agouti, pacas, armadillos and an opossum in Brazil suggest that a new species of *Theileria* may be infecting pacas, agouti and armadillos and a novel *Babesia* sp. may be infecting opossums [24]. *Hepatozoon* spp. are unique protozoan parasites as they are transmitted mainly through the ingestion of arthropods, which are the definitive hosts such as certain species of ticks and fleas [25, 26]. Criado-Fornelio et al. in 2009 identified *H. canis* as well as an unidentifiable *Babesia* sp. somewhat related to *T. equi* in capybaras from Brazil [27]. *Hepatozoon canis* has been previously identified in the canine population of Trinidad [28].

To date, there are no epidemiological data on which pathogens are present and the molecular characteristics of tick-borne pathogens in neotropical mammals in Trinidad. The health of captured wildlife is also not reported. As 75% of emerging pathogens are zoonotic and there has been increasing discovery of new tick-borne pathogens in humans, the objective of this study was to screen wildlife captured for human consumption for tick-borne pathogens belonging to the genera *Anaplasma*, *Babesia*, *Bartonella*, *Ehrlichia*, *Hepatozoon*, *Rickettsia* and *Theileria* using molecular methods.

## Methods

### Hunting

Hunting is legally permitted in Trinidad on an annual basis for 5 months beginning on the first day of October and ending on the last day of February. All species of neotropical animals screened in this study could be legally hunted during the 2017–2018 and 2018–2019 hunting seasons in Trinidad.

### Sample size estimates

An estimated 30,000 neotropical animals are harvested per year based on receipts submitted to the Ministry of Agriculture, Forestry Division [29]. The Cannon and Roe formula was used to estimate the minimum number of animals needed to detect one positive animal. Using an estimated prevalence of 5% for the detection of tick-borne pathogens and 95% confidence for detecting one positive animal, the estimated sample size using the simple binomial distribution was 59 [30].

### Sample collection

Samples were collected over two consecutive annual hunting periods. Any wildlife carcass submitted for necropsy at the veterinary diagnostic laboratory during this period was also included in the study. Each carcass was examined for ticks. The body condition score was noted for all the animals and assessed according to the criteria outlined in Table 1.

**Table 1:** Criteria used to assess the body condition score of each captured neotropical animal in this study Adapted and modified from Ullman-Culleré and Foltz [54]

Body condition	Fat stores	Muscling
Poor	Barely visible subcutaneous and abdominal fat	Bony prominences (hips, ribs, pin bone) highly visible
Adequate	Visible subcutaneous and abdominal fat without visual impairment of the organs	Bony prominences barely visible
Excessive	Subcutaneous and abdominal fat causing visual impairment of the organ	Bony prominences not detectable

EDTA blood samples were obtained from animals captured by hunters during the 2017–2018 and 2018–2019 hunting seasons. Global positioning system (GPS) coordinates for animals captured from each location were recorded and used to generate maps using the Free and Open Source QGIS software.

Three millilitres of whole blood was collected directly from the jugular vein or heart upon exsanguination of neotropical animals. A thin blood smear was made from each EDTA blood sample and stained with Wright-Giemsa for microscopic examination to detect tick-borne pathogens.

Animals were also examined for the presence of ticks. If ticks were present, they were stored and identified using morphological keys [2].

Additionally, a splenic tissue sample was obtained from any neotropical animal submitted to the necropsy laboratory of the School of Veterinary Medicine during the study period.

### DNA extraction

DNA was extracted from 100 µl of anticoagulated blood or 25 mg of splenic tissue using the Qiagen DNeasy Blood and Tissue kit (Qiagen, Maryland, USA) according to the manufacturer’s instructions. DNA concentrations were determined by measuring the absorbance at 260 nm (A260) with a NanoVue Spectrophotometer (GE Healthcare, UK Limited).

### Amplification of *Babesia* spp., *Theileria* spp. and *Hepatozoon* spp.

*Babesia*/*Theileria* (B/T) genera were amplified using primers RLBF2/R2, which amplifies a 540-bp fragment of the *18S rRNA* gene [31, 32]. *Hepatozoon* spp. could also be amplified using these primers to give a 400-bp product. Positive samples were further screened for *Babesia* spp. and *Hepatozoon* spp. DNA. The *18S rRNA* gene of *Babesia* spp. and *Hepatozoon* spp. was targeted using the primers Piroplasmid-F/Piroplasmid-R, which amplified a 350-bp fragment [33]. To identify cases of co-infection, positive samples were tested by an additional PCR assay using PIROA/PIROB primers, which amplified a 400-bp fragment of the *18S rRNA* gene of the *Babesia* spp. [34]. DNA from naturally infected dogs with *Babesia vogeli* (Bab9706) and another dog with *Hepatozoon canis* infection were used as positive controls. Amplified products were subjected to gel electrophoresis using 1.5% agarose in TAE buffer pre-stained with ethidium bromide and visualised under UV light.

### Amplification of *Bartonella* spp.

Real-time PCR was performed to amplify a 340-bp *gltA* gene fragment using primers 443F and 781R and 300-bp *ssrA* gene fragment using primers *ssrA*F and *ssrA*R (Table 2) with previously described reaction volumes and conditions [35]. DNA extracted from a culture of *Bartonella krasnovii* served as a positive control.

**Table 2:** Primers and their sequences used in this study

Primers	Sequence 5′-3′	Target gene	Target genera	Amplicon size (bp)	References
RLB-F2 RLB-R2	ACACAGGGAGGTAGTGACAAG CTAAGAATTTCACCTCTGACAGT	*18S rRNA*	*Babesia/Theileria*	540	[32]
Piroplasmid-F Piroplasmid-R	CCAGCAGCCGCGGTAATTC CTTTCGCAGTAGTTYGTCTTTAACAAATCT	*18S rRNA*	*Babesia/Hepatozoon*	350	[33]
PIROA PIROB	AATACCCAATCCTGACACAGGG TTAAATACGAATGCCCCCAAC	*18S rRNA*	*Babesia*	400	[34]
*16S*8FE B-GA1B	AGAGTTGGATCMTGGYTCAG CGAGTTTGCCGGGACTTYTTC	*16S rRNA*	*Ehrlichia/Anaplasma*	500	[37]
E.c *16S*-fwd E.c. *16S*-rev	TCGCTATTAGATGAGCCTACGT GAGTCTGGACCGTATCTCAG	*16S rRNA*	*Ehrlichia/Anaplasma*	123	[38]
EHR*16S*D EHR*16S*R	GTACCYACAGAAGAAGTCC TAGCACTCATCGTTTACAGC	*16S rRNA*	*Ehrlichia/Anaplasma*	345	[40]
443F 781R	GCTATGTCTGCATTCTATCA CCACCATGAGCTGGTCCCC	*gltA*	*Bartonella*	340	[35]
*ssrA*F *ssrA*R	GCTATGGTAATAAATGGACAATGAAATAA GCTTCTGTTGCCAGGTG	*ssrA*	*Bartonella*	300	[35]
rico173F rico173R	CGACCCGGGTTTTATGTCTA ACTGCTCGCCACTTGGTAGT	*gltA*	*Rickettsia*	133	[36]

### Amplification of *Rickettsia* spp.

Real-time PCR was also used to detect *Rickettsia* spp. by targeting a 133-bp fragment of the citrate synthase gene (*gltA*) using primers rico173F and rico173R (Table 2) as previously described [36]. DNA extracted from plasmid containing the rickettsial *gltA* gene served as a positive control.

### Amplification of *Anaplasma* spp. and* Ehrlichia* spp.

A portion of the *16S rRNA* gene of *Anaplasma*/*Ehrlichia* (A/E) genera was amplified by conventional PCR using primers *16S*8FE and BGA1B yielding a fragment of approximately 500 bp [37]. *Anaplasma marginale* was used as the positive control for this reaction. Samples were also subjected to a real-time PCR assay that amplified *Anaplasma* and *Ehrlichia* DNA using the primers E.c *16S*-fwd and E.c. *16S*-rev, which amplified a 123-bp fragment of the *16S rRNA* gene as previously described [38]. Samples were considered positive for *E. canis* DNA when the cycle threshold values were in the range of 25–40 and the melting curves were identical to those of the positive control [39]. DNA extracted from *E. canis* cell culture was used as the positive control. Positive samples from the real-time PCR reaction were further analysed by conventional PCR using primers EHR*16S*D and EHR*16S*R to amplify a 345-bp fragment of the *16S rRNA* gene of the genera *Anaplasma* and *Ehrlichia* using the previously described reaction volumes and conditions [40]. PCR products were electrophoresed on 1.5% agarose gels stained with ethidium bromide and visualised under UV light.

DNA from blood of an uninfected dog was used as a negative control. In addition, non-template controls (NTC) as well as positive controls were included in each reaction in duplicate. Non-template control reactions were done using the same procedures and reagents as described above but without DNA added to the PCR reaction to rule out PCR contaminations and nonspecific reactions. Sequences of each primer and the targeted genera are given in Table 2.

### Sequencing

Amplicons at 400–500 bp (A/E) and 460–520 bp for B/T were sequenced at Macrogen Inc. (Seoul South Korea), and those at 345 bp for (A/E) PCR were sequenced at Hy Laboratories Ltd. (Rehovot, Israel). Sequences were compared to those deposited in GenBank using the Basic Local Alignment Search Tool (BLAST) [41]. A result was considered positive for a certain pathogen DNA if there was at least a 97% nucleotide identity with a known GenBank accession number. Three of these sequences were deposited in GenBank databases with accession numbers MZ506884, MZ506850 and MZ506838.

## Results

### Sample collection

**Fig. 1:**
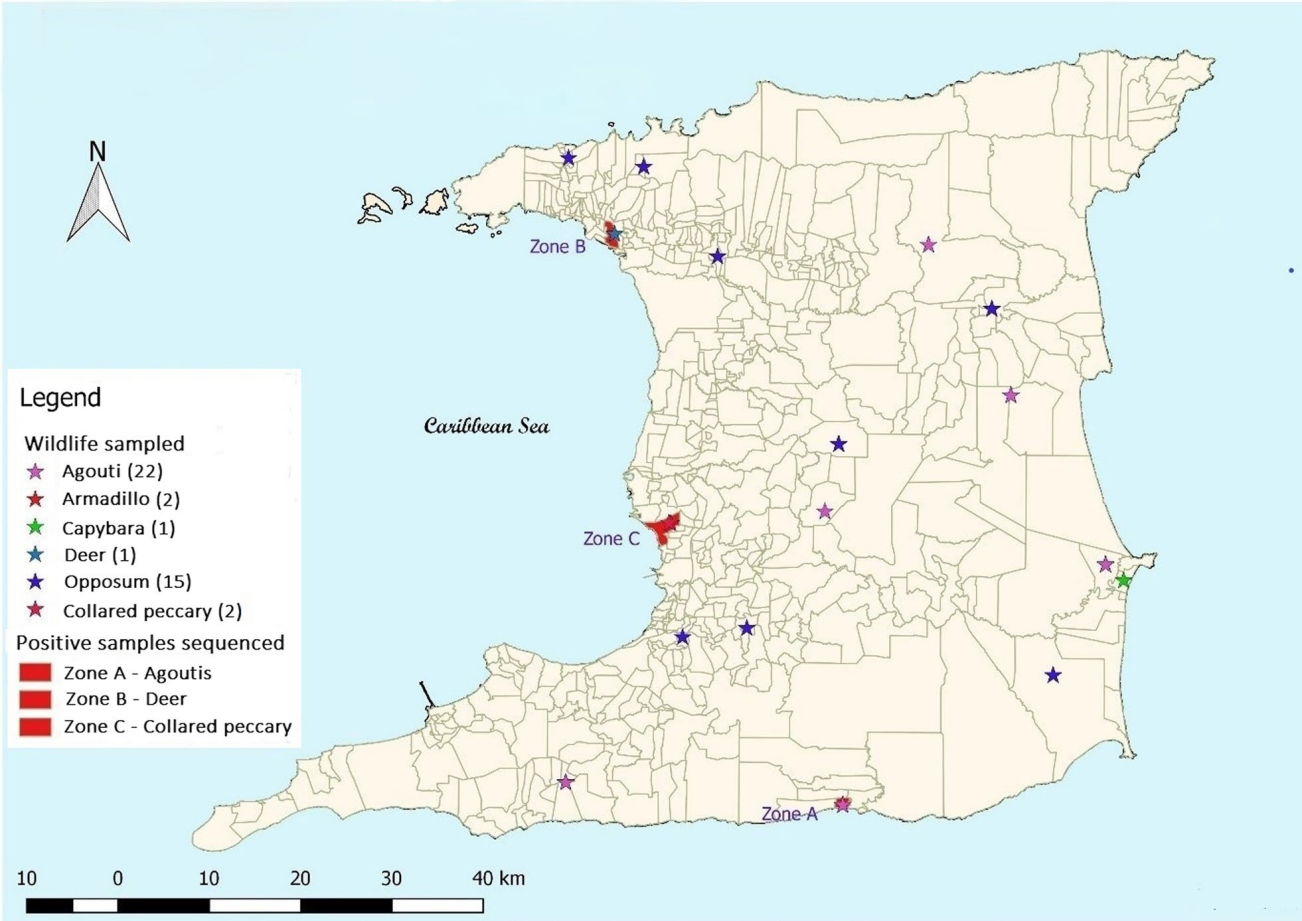
Map of Trinidad showing sampling sites Map generated using the free and open source QGIS software

A total of 43 blood samples were collected from various neotropical mammalian species over two consecutive hunting seasons from different locations throughout Trinidad (Fig. 1). The majority of the samples collected were from agoutis (*n* = 22) in this study, followed by opossums (*n* = 15), collared peccaries (*n* = 2), nine-banded armadillos (*n* = 2), red brocket deer (*n* = 1) and capybara (*n* = 1). Two neotropical carcasses (red brocket deer and capybara) were submitted for post-mortem examination during the study period. Splenic samples obtained from these two carcasses were included in this study.

The body condition of all animals harvested was classified as adequate and no untoward clinical manifestations of disease were reported. The red brocket deer included in this study was rescued by villagers who found the animal in distress near a roadway. The animal was severely infested with ticks, which were predominantly attached to the ears, had pale mucous membranes and voided brown urine. An attending veterinarian reported the deer to be in respiratory distress and it was euthanized. The bladder contained red-brown, slightly opaque urine. There were no significant findings on necropsy of the capybara carcass.

### Microscopic examination of blood and splenic smears

**Fig. 2:**
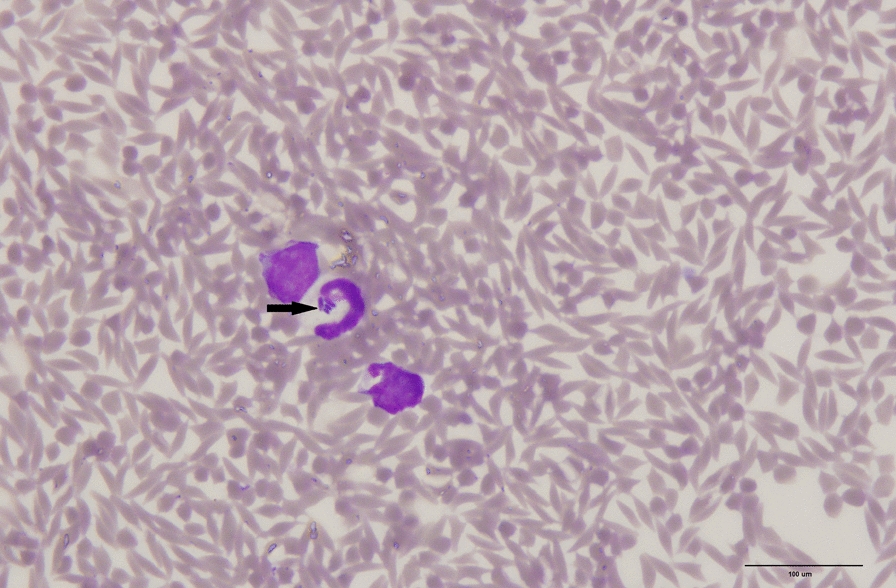
Basophilic bacterial rod-like inclusions (arrows) observed in the cytoplasm of a neutrophil in the blood. Scale bar represents 100 µm

Protozoan parasites were not detected on microscopic examination of all of the blood smears; however, examination of the blood smears from the red brocket deer revealed basophilic bacterial rod-like structures in the cytoplasm of neutrophils. These bacterial rod-like structures were observed in approximately 1 out of every 80 neutrophils. These structures were also observed in the neutrophils of impression smears of the spleen (Fig. 2). Ten (23%) blood smears also contained occasional bacterial cocci or rods that were visualised extracellularly, indicating probable contamination.

### Tick identification

Ticks were recovered from one animal (a red brocket deer) and identified as *Haemaphysalis juxtakochi.* Ticks were not observed on any other animals sampled.

### Molecular analyses

The results for the three PCR protocols (RLB F2/R2, Piroplasmid F/R, PIROA/PIROB) used to evaluate the *18S rRNA* gene were in agreement. DNA for *Babesia/Theileria* spp. was only amplified for two adult female agoutis. All other samples were negative. The sequences obtained from the two agoutis were more similar to *Theileria* than to *Babesia* spp., with 90.4% homology to and 99% query coverage of *Theileria* sp. OT3 isolate (GenBank: MG930118) from a goat in China.

Two samples (DNA extracted from blood collected from the collared peccary and spleen of the red brocket deer) yielded amplified products of approximately 500 bp, 123 bp and 325 bp from amplification of the *16S rRNA* gene using primers 1*6S*8FE and B-GA1B, E.c. *16S*-fwd & E.c. *16S*-rev and EHR*16S*D and EHR*16S*R, respectively. All samples were negative for *Bartonella* and *Rickettsia* DNA.

The sequence obtained from DNA extracted from blood of the collared peccary using primers E.c. *16S*-fwd and E.c. *16S-*rev had 100% nucleotide identity to uncultured *Ehrlichia* spp. (GenBank: MF153980) and 98.8% nucleotide identity to *E. canis* (GenBank: MT066094) with 98% query coverage for both. Sequence of the amplified product obtained from the splenic tissue of the deer using primers *16S*8FE/BGA1B revealed 99.8% nucleotide identity to *A. bovis* sequences from a marsh deer (*Blastoceros dichotomus*) in Brazil (GenBank: JF952893) and 97.5% nucleotide identity to an *A. bovis* sequence obtained from a goat in China (GenBank: MH255939).

## Discussion

The neotropical mammalian wildlife of Trinidad has long remained unexplored with respect to production and scientific research. However, within recent years there has been an increase in neotropical animal production as their contributions toward employment, food security and conservation needs of humans are being realised [7]. Wildlife-associated infectious diseases are also becoming increasingly important because of the rise in interaction of these species with humans, domestic animals and livestock. This is the first report on the use of molecular methods to screen tick-borne pathogens in neotropical animals in Trinidad. As little is known about the infectious agents present in the neotropical animal population, the findings uncovered in this study are of major importance to elucidate the pathogens that may have a zoonotic potential.

*Theileria equi* found in equids is the only *Theileria* spp. reported in Trinidad [42, 43]. BLAST analyses showed that the sequences obtained from the amplified products of the two agoutis were more similar to *Theileria* spp. than *Babesia* spp. It should also be noted that the two sequences were not closely related to *T. equi* from Trinidad as they had 92.3% nucleotide identity to and 71% query coverage of *T. equi* sequences from Trinidad (Genbank: KY053284 and KY053283). The results from this study suggest that another *Theileria* sp. may be present in the wildlife population of Trinidad and has to be further investigated. Although *Hepatozoon* spp. have been previously reported in capybara and paca, it has not been widely reported in neotropical animals. Furthermore, it was not detected in this study, which is in agreement with other published reports. *Hepatozoon* species-specific primers were not used; however, the fragment generated by *Hepatozoon* DNA is 400 bp while that of *Babesia /Theileria* DNA is 540 bp. Further work using a larger sample size is needed. Criado-Fornelio et al. [44] did not detect *Hepatozoon* spp. in a sample of 15 opossums in Brazil. A novel *Hepatozoon* species was identified from the lung tissue of a lowland paca in Brazil; however, that study failed to detect *Hepatozoon* DNA in samples obtained from nine-banded armadillos (*n* = 32), agoutis (*n* = 2), collared peccaries (*n* = 11), opossums (*n* = 19) and red brocket deer (*n* = 3) [24].

Deer have been identified as important reservoirs of *Anaplasma* spp. and *Ehrlichia* spp. including *A. bovis* [45]. Stressors such as poor nutrition, handling, harsh weather or environmental conditions may result in clinical manifestations of tick-borne disease [46]. The hard tick, *Haemaphysalis juxtakochi*, parasitise deer, paca and the collared peccary and is associated with the transmission of several *Rickettsia* spp. [47]. There is limited information on the reservoir hosts and tick vectors of *E. chaffeensis* on the South American continent [48]. This tick-borne pathogen was also reported in the marsh deer (*Blastocerus dichotomus*) population in Brazil and Argentina [48]. The spleen of the deer was positive for *A. bovis* DNA based on molecular analysis; however, no DNA of this organism was amplified from the blood. The reason for this could be that the level of bacteraemia was below the PCR assay's sensitivity and/or the organism may have been sequestered in the spleen and not present in the central circulation [49]. The spleen removes aged or parasitised blood cells and causes haemoconcentration of blood, which may account for the detection of *Anaplasma* sp. DNA only in the splenic tissue. This study also demonstrated that splenic tissue, in addition to whole blood samples, should be harvested to increase the sensitivity of detection of tick-borne pathogens of neotropical animals using molecular methods, as previously shown for domestic animals [49, 50].

The identification of DNA of *Bartonella* spp. directly from the blood of animals or humans using molecular techniques can be challenging as it lacks sensitivity even in the presence of bacteraemia [51]. This is possibly one of the reasons for the negative PCR results obtained in this study. Some researchers recommend that suspected samples should undergo an enrichment culture before the PCR amplification to improve the sensitivity in detecting *Bartonella* spp. from the blood of animals and humans [51, 52]. Using whole blood is less sensitive than using tissue samples for the detection of *Rickettsia* spp. when there are low numbers of bacteria circulating in the blood, especially in the absence of advanced disease and severe infections [53].

The number of samples and species collected in this study were completely dependent on the participating hunters and their preferred species to hunt. Hunters were reluctant to participate in this study, restricting the number of collected samples. Trinidad also experienced persistent torrential rainfall and severe flash flooding in its Northern, Central and Southern regions during the study period. Due to this inclement weather and the terrain conditions thereafter, data collection was postponed for several months until participating hunters found conditions to be safe and accessible for hunting. This unexpected and prolonged period of inactivity greatly impacted the number of samples collected during this study.

The case of the red brocket deer shows that hunters will encounter animals of varying health and animals with ticks. The red brocket deer might have been consumed if this chance intervention by villagers had not occurred. Hunters must be educated on recognising normal and abnormal animals and should be aware of the potential risks associated with contact with wildlife and carcasses infested with ectoparasites. Ten blood smears contained occasional extracellular bacterial cocci or rods, which may be attributed to bacterial contamination during the slaughter process. This incidental finding is of public health significance as it indicates that slaughter practices may not be hygienic, leading to haematological spread of bacterial contaminants and cross contamination of surfaces. These factors can lead to a reduction in wholesomeness of the carcass and highlights the potential for a food safety hazard. Therefore, hunters should also be trained in safe animal slaughter procedures, including cleaning tools between butchering to avoid cross contamination. With the broad gaps of knowledge surrounding Trinidadian wildlife, this study serves as a starting point to encourage further investigations into the health of the neotropical population of the rainforest, which has a very close relationship with Trinidadian society and culture.

## Conclusion

The present study confirmed the presence of tick-borne pathogens in wildlife captured for human consumption in Trinidad, including *A. bovis* in a deer, a *Theileria* sp. in agoutis and an *Ehrlichia* sp. (related to *E. canis*) in a collared peccary. Further research is needed to investigate the identity of these species and their medico-veterinary significance.

## Data Availability

The data supporting the conclusions of this article are included within the article. Raw data can be shared with researchers upon a specific request.

## Abbreviations

A/E: *Anaplasma/Ehrlichia*
B/T: *Babesia/Theileria*
bp: Base pairs
BLAST: Basic Local Alignment Search Tool
DNA: Deoxyribonucleic acid
EDTA: Ethylenediaminetetraacetic acid
GPS: Global positioning system
NTC: Non-template control
rRNA: Ribosomal ribonucleic acid
QGIS: Quantum Geographic Information System
TAE: Tris–acetate-EDTA
UV: Ultraviolet
